# The ups and downs of a canopy-forming seaweed over a span of more than one century

**DOI:** 10.1038/s41598-019-41676-2

**Published:** 2019-03-27

**Authors:** Aurélie Blanfuné, Charles François Boudouresque, Marc Verlaque, Thierry Thibaut

**Affiliations:** Aix-Marseille University, Mediterranean Institute of Oceanography (MIO), CNRS/INSU, IRD, UM 110, Campus of Luminy, 13288 Marseille, cedex 9 France

## Abstract

Canopy-forming seaweeds constitute marine forests that deliver ecosystem services. The worldwide range shift, sharp decline or loss of many of these forests, caused by the cumulative impact of increasing human pressure and climate change, have been widely documented. Contrasting examples, reflecting higher than expected resilience, have been more rarely reported. Here, we took the opportunity of having at our disposal a two-century suite of documents (herbarium vouchers, articles) and a ~120-year observation period, dealing with a long-lived brown seaweed, *Cystoseira mediterranea*, along a well-explored Mediterranean coastline in the Gulf of Lions, to depict the fate of its populations. In addition, we provided baselines for future surveys, with a high degree of accuracy. The northernmost population, scattered on rare suitable substrates, gradually declined and has been extinct since the 1980s. The length of shore occupied by the southern population showed a long-term decline trend, with two sharp minima followed by partial recovery. The causes of the decline differ between sites and periods: coastal development, pollution, competition with mussels, heatwaves and exceptional storms. Overall, the Gulf of Lions populations reflects long-lasting resilience, higher than expected, and a health status that is better than that reported for many other canopy-forming seaweeds.

## Introduction

Canopy-forming seaweeds, generally large brown algae such as kelp and fucoids, dominate shallow rocky coasts of the world’s temperate and cold-water seas^1^, providing high levels of delivery of ecosystem services (e.g.^1–3^). Despite differences in the dominant species, canopy-forming seaweeds worldwide share some common features in their structure and functioning. They influence their environment and other organisms, thereby functioning as “ecosystem engineers” (sensu Jones *et al*.^4^), by altering e.g. light^5^, water flow^6^ and sedimentation^7^. They play a pivotal role in coastal areas: biomass, primary production, nurseries, etc. (e.g.^2^).

The ecosystems they form are suffering a global decline caused by the cumulative impact of increasing human pressure acting over time and in unison (e.g. destruction of habitats, pollution, non-indigenous species, overfishing, damage by fishing nets, overgrazing due to extirpation of predators of grazers, aquaculture and global warming) (e.g.^2,8–15^). Natural recovery after dramatic decline has been observed mostly for short-lived and/or high dispersal capacity species (e.g. loss and recovery of the giant kelp *Macrocystis pyrifera* (Linnaeus) C. Agardh due to the fluctuations of sea-urchin and otter populations, 14; recovery of seaweed forests after the 2011 Japanese tsunami)^15^. In order to understand the magnitude and pattern of marine ecosysytem shifts, long-term descriptive data (multidecadal data series) are paramount for understanding these phenomena and for acquiring the baseline^16,17^.

In the Mediterranean Sea, cases of regional loss and local extinction of canopy-forming species (Fucales, Phaeophyceae) are numerous, leading to a shift to less structurally complex communities, dominated by turf-forming, filamentous or other ephemeral seaweeds (e.g.^18–31^). Most of the species losses concern sublittoral species, which can be controlled mainly by a top-down mechanism^27,32,33^. Reports of recovery of Fucales populations in the Mediterranean are rare, and even in Marine Protected Areas (MPAs), the complete loss of Fucales populations has been reported^19,23,29,34^. Only two species bearing aerocysts, *Cystoseira compressa* (Esper) Gerloff & Nizamuddin subsp. *compressa* and *Sargassum vulgare* C. Agardh, have been recorded as remaining abundant in many regions, whatever the anthropogenic impact (e.g.^22,35,36^), and able to recover after depletion^37,38^. Among the Mediterranean Fucales, the long-lived species of the genus *Cystoseira* C. Agardh (between one and more than five decades^39,40^) are the main component of the marine seaweed forests from the sea-surface down to 40 m depth. Most of the taxa have strong ecological constraints that limit their distribution to particular and narrow habitats, which therefore render their recovery difficult after depletion.

Among the few taxa strictly restricted to dwelling close to sea level on moderately to strongly exposed rocky coasts, *Cystoseira mediterranea* Sauvageau is a long-lived brown alga described from Banyuls-sur-Mer (French Catalonia)^41^, restricted to the Mediterranean Sea^42,43^ and the immediate vicinity of the Gibraltar Strait on the Atlantic Morrocan coast^44,45^. It is a single axis plant up to 40–50 cm height that forms extensive photophilous stands on shallow and wave-exposed rocky substrates, tolerating regular emersion caused by wave-movements and tides. Generally, the species does not thrive deeper than 0.5–1.0 m below the mean sea level, in a sea where the tide range is extremely low^46^. It is a perennial species: the axis is present year-round, while the branches are deciduous. *Cystoseira mediterranea* is one of the most productive Mediterranean seaweeds, with a marked vegetative seasonal variation^47,48^. The species is considered to have a high ecological status within the framework of the EU (European Union) Water-Framework Directive (2000/60/EC) (e.g.^31,49,50^). Pollution and eutrophication have a clear negative impact on the coverage of *C*. *mediterranea*^48^.

The aim of this study was (i) to provide an exhaustive map of the current distribution of *C*. *mediterranea* over the entire French Mediterranean coast; (ii) to compare this distribution with historical data to assess losses, gains or stability; (iii) to identify and analyze the dynamic of the populations and the putative causes of fluctuation of its abundance.

## Material and Methods

### Study area

**Figure 1:**
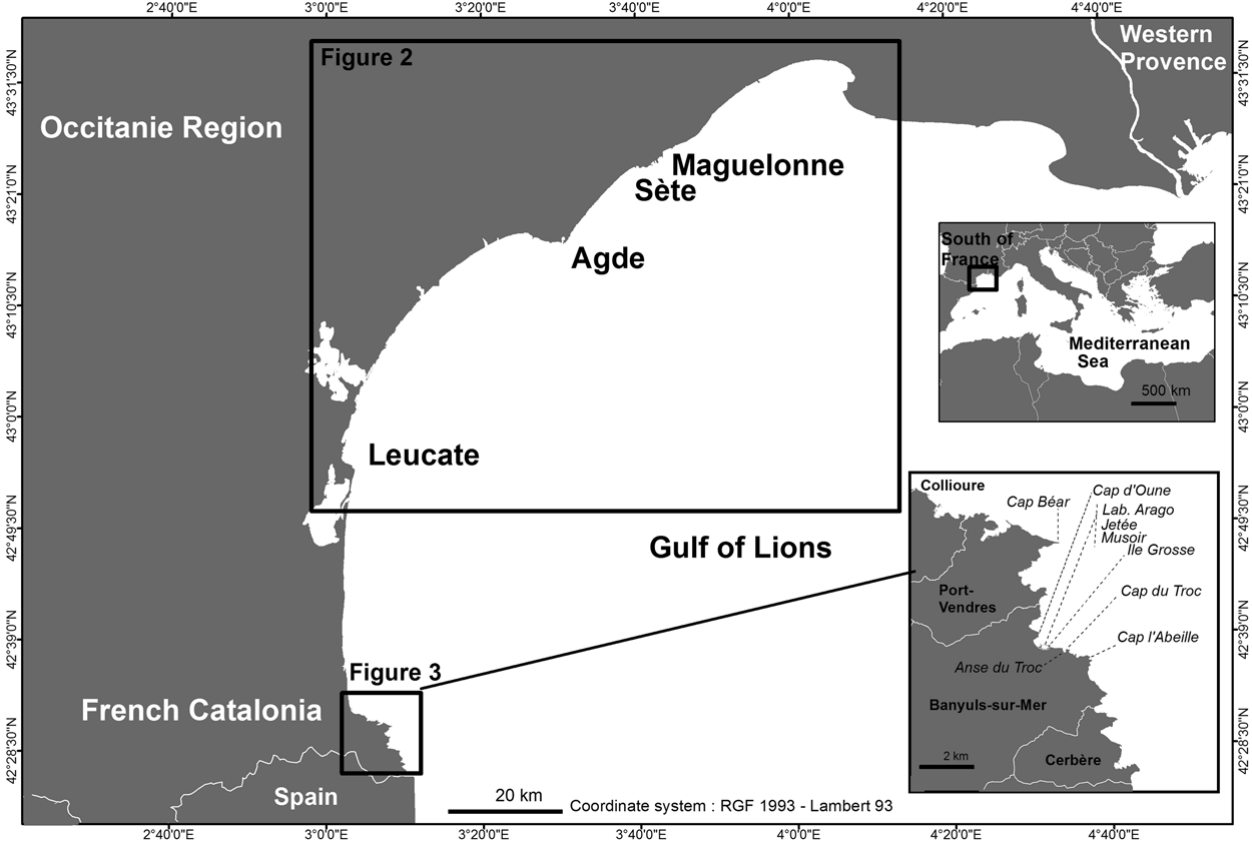
Study area along the French Mediterranean coast. Boxes: see Figs 2 and 3.

We investigated the entire French Mediterranean coast where *Cystoseira mediterranea* lives, corresponding to the *Occitanie* Region in the Gulf of Lions (Fig. 1). The species is absent from western Provence, eastern Provence, the French Riviera (including the Principality of Monaco) and Corsica, where it can be replaced on hard substrates by the vicariant species *C*. *amentacea* (C. Agardh) Bory^20^. *Cystoseira mediterranea* is also abundant along the Spanish Catalonian coast^47^. The coast of the Gulf of Lions is mainly sandy, and only a few locations, namely Sète (3.3 km of rocky coasts), Agde (9 km), Leucate cliffs (2.5 km) and French Catalonia (58 km) are able to host *C*. *mediterranea*. These locations are isolated from each other by tens of kilometers of sandy substrate.

### Historical data in the study area

Historical French records of *C*. *mediterranea* were analysed from published articles, unpublished reports and herbarium vouchers. Printed documents are kept in the ‘Macrophyte research platform’ of the Mediterranean Institute of Oceanography (Aix-Marseille University, France).

Voucher specimens held in herbaria are an exceptional source of data, allowing verification of the identification of the specimens. We surveyed 3 821 voucher specimens held at the following Institutes (Herbaria acronyms after Thiers^51^): *Muséum d’Histoire naturelle d’Aix-en-Provence* (AIX), *Muséum d’Histoire naturelle d’Avignon* (AV), *Herbarium of University of Coimbra* (COI), *Herbarium of Göteborg* (GB), *Mediterranean Institute of Oceanography – Aix Marseille University* (HCOM), *University of Girona* (HGI), *Musée botanique cantonal de Lausanne* (LAU), *herbarium of Lund* (LD), *University of Montpellier* (MPU), *Herbarium Oskarshamn* (OHN); *Muséum National d’Histoire naturelle de Paris* (PC), *Herbarium Stockholm* (S), *Muséum d’Histoire naturelle de Toulon* (TLON), *Herbarium Umeå* (UME), *Herbarium Uppsala* (UPS), *Botanical garden of Villa Thuret*, *Antibes* (VTA), *Muséum d’Histoire naturelle de Marseille*, *Musée Océanographique de Monaco*, *Nice-Sophia Antipolis University*, and *Herbier du Hamas de J*.*H*. *Fabre*. Some of these herbaria data are available through the Sweden’s Virtual Herbarium (http://herbarium.emg.umu.se/).

The specimens of *Cystoseira mediterranea* were either correctly labelled, in Herbaria, or misidentified as *C*. *mediterranea* var. *valiante* Sauvageau, *C*. *ericoides* (Linnaeus) C. Agardh and *Fucus selaginoides* Linnaeus. For each specimen, the following data were noted: locality, date of collection, taxonomic name used and reference number. The misidentifications were corrected.

### Field work

The distribution pattern of *C*. *mediterranea* in the Gulf of Lions was investigated by field surveys in April 2007 and 2012, on all the possible suitable substrates (very shallow rocky substrates from the mean sea level down to less than 1 m depth) for the species (Fig. 1).

Spring was chosen because primary branches of *C*. *mediterranea* are then fully developed. The method (hereafter) and the observer were the same as in 2003^19^.

*Cystoseira mediterranea* populations were recorded on black and white A3 format aerial photographs from the IGN (French National Institute of Geographical and Forest Information: BD Ortho); Google Earth® was also used. The scale was 1:2 500. Three people were on board a small boat (length 5 m) moving at low speed (3 to 6 km.h^−1^/1.6 to 3.2 knots) a few meters offshore, along 72.8 km of shoreline. *Cystoseira mediterranea* populations were recorded within four classes: C 0 = absent, C 1 = scattered individuals, C 2 = patches of dense stands, C 3 = almost continuous or continuous belt. Furthermore, for each 50-m sector, corresponding to a class of *C*. *mediterranea* (C 0 through C 3), we also recorded when present the dominant taxa and functional groups other than *C*. *mediterranea*, e.g. articulated corallines (mainly *Corallina caespitosa* R.H.Walker, J.Brodie & L.M.Irvine), algal turf and the mussel *Mytilus galloprovincialis* Lamarck, 1819. Overall, the exhaustive exploration of the coastline was summarized in 1 456 sections 50-m long.

Doubtful specimens were collected for checking in the laboratory using the appropriate litterature^52,53^. Voucher specimens are deposited in Herbarium Thibaut (HCOM) held at the Mediterranean Institute of Oceanography - Aix-Marseille University.

### GIS analyses

Each location (past or present) of *C*. *mediterranea* was geo-localized and the past and present distribution patterns were analyzed on a GIS (Geographical Information System) database (ArcGis10®).

## Results

**Figure 2:**
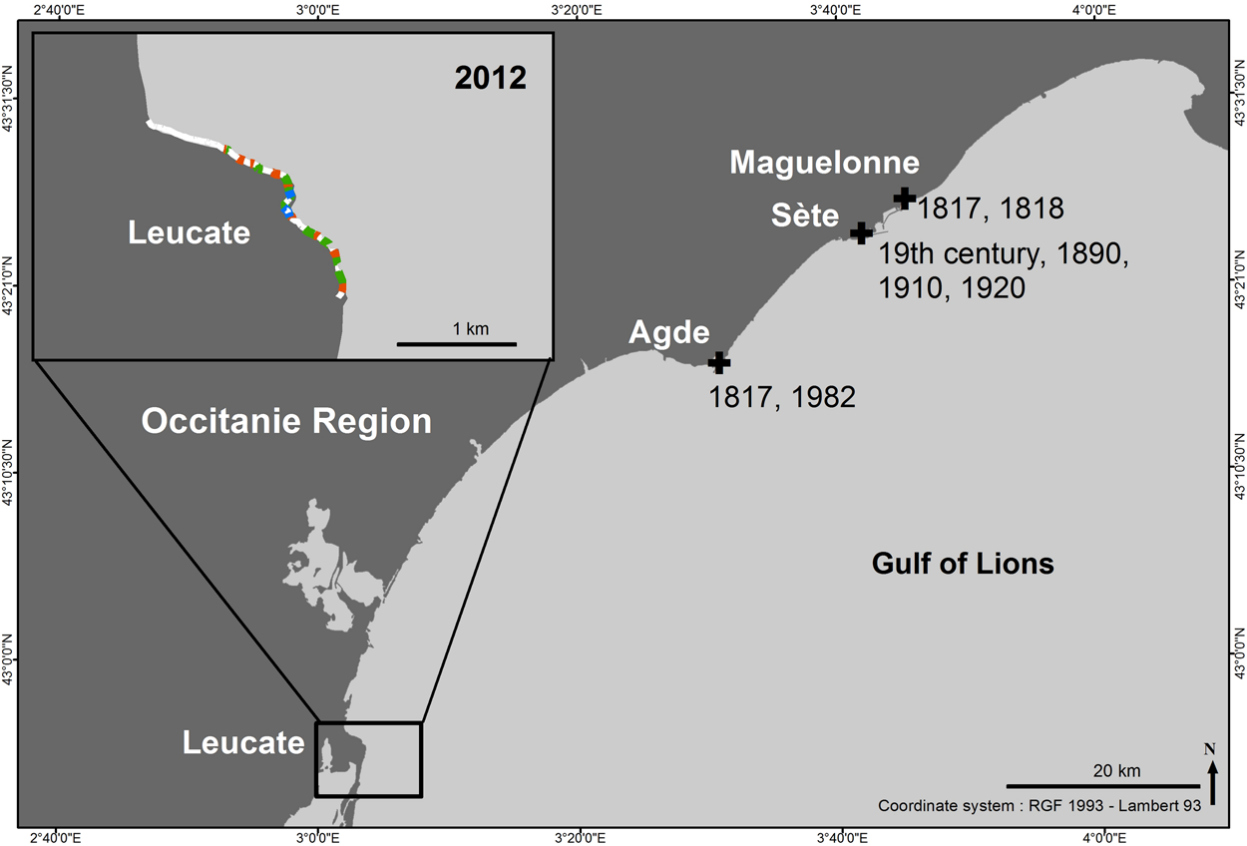
Distribution of *Cystoseira mediterranea* along the coastline of the *Occitanie* Region. Black crosses = Historical records of *C*. *mediterranea* (data from vouchers and literature; see Table S). Within the inset closeup of the Leucate area: white = absent; orange = scattered individuals (class C1); green = patches of dense stands (class C2); blue = continuous or almost continuous belt (class C3).

**Figure 3:**
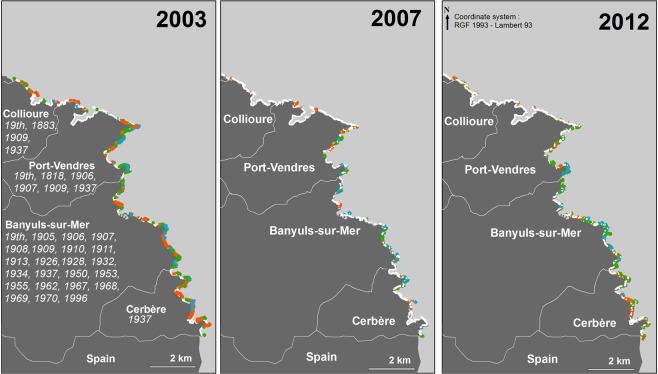
Distribution of *Cystoseira mediterranea* along the coastline of French Catalonia. Dates of historical records of *C*. *mediterranea* (from vouchers and literature; see Table S1). White = absent; orange = scattered individuals (class C1); green = patches of dense stands (class C2); blue = continuous or almost continuous belt (class C3).

A total of 87 historical records of *Cystoseira mediterranea* have been found from the 19^th^ (1817 for the oldest, at Agde) to the 20^th^ centuries; 19 in the *Occitanie* Region (Sète and Agde) and 68 in French Catalonia (Figs 2 and 3, Table S1).

### Sète

The most ancient specimens of *C*. *mediterranea* were collected in 1817 and 1818, cast ashore from the vicinity of Sète (at Maguelonne), then at Sète, mainly on the harbour jetty from 1890 to 1920; 1920 is the last record of the species at Sète. In April 2007 and 2012, all suitable habitats (about 10 km of coastline) were thoroughly explored: *C*. *mediterranea* was absent everywhere and its habitat was mainly colonized by the articulated coralline *Corallina caespitosa* and the mussel *Mytilus galloprovincialis* (Fig. 2 and Table S1**)**.

### Agde

The species was reported and collected from 1817 to its last record in 1982. Lauret^54^ noted that the species was rare at Agde, only located in one place at La Grande Conque, hampered by heavy sedimentation and outcompeted by *Corallina caespitosa* and *Mytilus galloprovincialis*. As in Sète, in April 2007 and 2012, all suitable habitats were thoroughly explored (about 7 km of coastline): *Cystoseira mediterranea* was everywhere absent and its habitat was mainly colonized by *Corallina caespitosa* and *Mytilus galloprovincialis* (Fig. 2 and Table S1).

### Leucate cliffs

No historical data was found for the rocky coast of Leucate. This 2.5 km long coastline consists of natural substrate, of which 1.18 km was colonized by *C*. *mediterranea* (2012 field survey): continuous or almost continuous belt (1.9%), patches of dense stands (25.7%) and scattered individuals (14.7%), but this was unlikely to be a recent colonization, more probably a hitherto unexplored shore due to its difficult access.

### French Catalonia

Along this rocky coast, the length of natural rocky substrates and the man-made hard substrates represent 56.6 km and 1.4 km, respectively. The oldest record of *C*. *mediterranea* dates back to 1818 at Port-Vendres. Subsequently, this stretch of shore has been thoroughly explored by phycologists, especially since the establishment of a marine laboratory at Banyuls-sur-Mer in 1872. Despite fluctuations in abundance and local losses (see below), the species has been continuously present in the area (Fig. 3, Table S1).

Between 2003^19^, 2007 and 2012 (present study), marked fluctuations in the abundance of *C*. *mediterranea* were recorded (Table 1). Between the three survey dates, the presence and abundance of *C*. *mediterranea* and of other dominant species conspicuously changed (Fig. 3, Table 1). *Cystoseira mediterranea* experienced a severe decline in 2007, and then partly recovered. At the same time, the abundance of the mussel *Mytilus galloprovincialis* decreased, while that of the articulated coralline *Corallina caespitosa* expanded. In 2003, the populations of *C*. *mediterranea* were sometimes mixed with mussels, while in 2007 and 2012, *C*. *mediterranea* and mussel stands were situated side by side, rather than mixed. Such sharp fluctuations cannot be explained by survey biases, since the survey method was exactly the same, the exploration of the shore exhaustive and the field team headed by the same investigator (TT).

**Table 1:** Percentage of the rocky shoreline occupied by *Cystoseira mediterranea* (three classes of abundance) and, when absent, dominated by articulated corallines (mainly *Corallina caespitosa*), *Mytilus galloprovincialis* and algal turf, along the rocky coast of French Catalonia, in 2003, 2007 and 2012. 2003: computed from the unpublished field database of Thibaut *et al*.^19^.

	2003	2007	2012
*Cystoseira mediterranea* (all classes of abundance)	32.9	9.5	20.1
C1: scattered individuals	15.8	2.8	5.2
C2: patches of dense stands	12.3	3.6	10.9
C3: continuous or almost continuous belt	4.8	3.1	4.0
Articulated corallines	3.2	3.3	41.7
*Mytilus galloprovincialis* (mussels)	24.3	27.6	16.2
Algal turf	39.6	59.6	22.0

## Discussion

Since the loss of the Sète and Agde populations, Leucate represents the new northernmost limit of *Cystoseira mediterranea* in the Mediterranean Sea, which represents a 68 km withdrawal to the south.

The cause of the local extinction is clearly linked to the destruction of the habitat at Sète: the population was recorded on the harbour jetty, which has been rebuilt and extended several times since 1920^55^. Furthermore, there has been a marine research centre at Sète since 1879, and after 1920 the species was never recorded again, whereas intensive phycological sampling was carried out during the 20^th^ century at this site. At Agde, the species was never recorded after 1989. The loss of *C*. *mediterranea* in this area could also be explained by habitat destruction, as most of the coastline has been urbanized, the cliff of ‘La Grande Conque’ was dynamited in the 1990s, and the remaining rocky reefs have been strongly worn by sand action during storms (Michel Lauret pers. com.). From 1969 to 1992, the coast was greatly altered, with the extension of the harbour and the construction of breakwaters^55^. Habitat destruction is regarded as one of major causes of the disappearance of several very shallow-dwelling *Cystoseira* species such as *C*. *amentacea* and *Cystoseira crinita* Duby^20,30^. At Sète and Agde, we can consider that *Cystoseira mediterranea* is extinct, while the natural recovery of *C*. *mediterranea* seems unlikely because of its life-history traits (non-floating species, low egg dispersal). If the rare event of long-distance dispersal via entangled floating rafts has been documented for its vicariant *C*. *amentacea*^20^, no floating rafts can spread seeds along the coasts of Sète and Agde as the species has never been recorded in Provence, along the French Riviera, or along the coasts of the Ligurian and Thyrrenian Seas, and the Northern Mediterranean Current flows southwards in this area^56,57^, which hampers possible recolonization from southern localities. Although *C*. *mediterranea* was already very uncommon at Agde and Sète due to the rarity of suitable (generally man-made) substrates, and the loss of these two populations was mainly due to the destruction of these substrates, the extinction of the species at its northernmost localities represents one of the outstanding results of the study.

**Figure 4:**
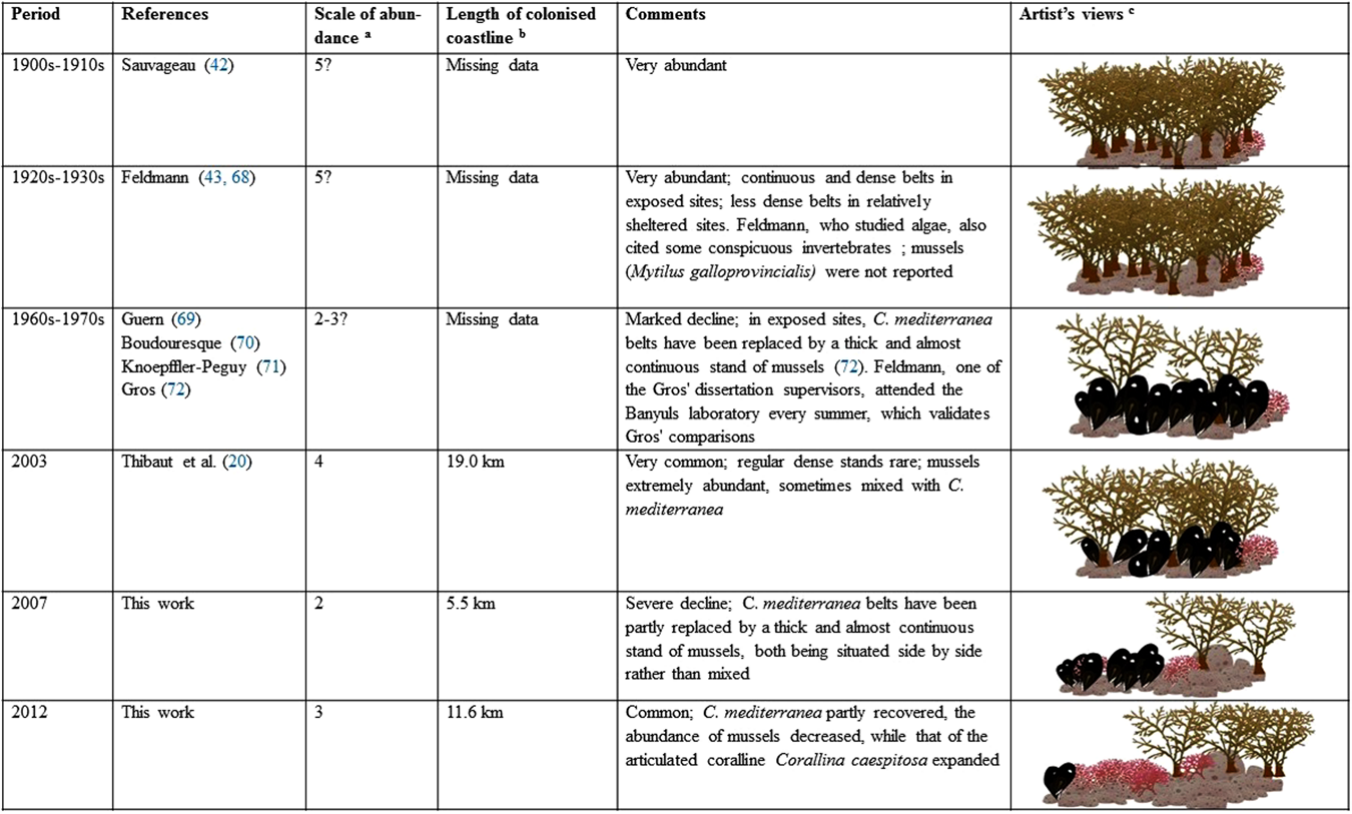
Changes over time in the abundance of *Cystoseira mediterranea* in French Catalonia, from Collioure to the Spanish border, with comments on *Mytilus galloprovincialis* populations. ^a^Semi-quantitative scale of abundance: 5 = Very abundant; all suitable habitats are occupied. Non-occupied suitable habitats represent a negligible length of the coastline. 4 = Abundant. Non-occupied suitable habitats represent a small but measurable length of the coastline. 3 = Discontinuous stands, occupying less than 50% of the suitable habitats. 2. Discontinuous stands, occupying less than 25% of the suitable habitats. 1 = Scattered stands and individuals, occupying less than 5% of the suitable habitats (not recorded). 0 = absent (not recorded). ^b^Length of coastline measured on a map at a scale of 1/2 500. The rocky coast measures 38 km. ^c^Artist’s views: original drawings.

Are the fluctuations observed along the coasts of French Catalonia a recent phenomenon or phases of cyclical events? *Cystoseira mediterranea* is a long-lived species, easy to observe in its quite shallow habitat, even without diving or snorkeling, and the area has been more or less continuously visited by phycologists over one century. We therefore attempted a reconstruction of the putative long-term changes in *C*. *mediterranea* abundance (Fig. 4). Two minima can be established, with reasonable certainty: 1960s-1970s and 2007, the latter being the most severe. Other minima could have occurred in the past, although they have gone unnoticed. Overall, the general trend over the past century has been a decline. However, in contrast with many stands of long-lived brown algae worldwide, with a steady, drastic, unidirectional decline (e.g.^9,10,58^), the decline of the *C*. *mediterranea* populations of French Catalonia has been marked by clear-cut ups and downs.

The rapidity of the 2012 natural recovery, just 5 years after the previous minimum, was unexpected. Long-lived seaweeds, especially fucoids, are often K strategists, with heavy eggs disseminated at short distances and a low rate of recruitment^59–63^. This dynamic, with decline and recovery trajectories, has been previously observed for kelp forest (Laminariales such as *Laminaria* and *Macrocystis*) around worldwide^2,14,64^, in contrast to the sharp decline observed for fucoids in the Mediterranea Sea. This contrasting pattern could be due to the single generation life history, with low dispersal of propagules, vs. two generations and long distance dispersal, respectively. In addition, recolonization can be hampered by the replacement stand (here, by mussel and articulated coralline assemblages), independently of the availability of propagules^65,66^. Similar relatively rapid recovery of *Cystoseira* populations has already been documented, e.g. in Croatia (Adriatic Sea), with a recovery time of ~10 years^37^.

A possible cause for the observed changes in the distribution and abundance of *C*. *mediterranea* could be the anthropogenic impact. Pollution, port facilities and coastal development are blamed as a cause of the decline of canopy-forming seaweed worldwide^9,67^. These factors are probably responsible for the decline of the range of *C*. *mediterranea*, with its disappearance at its northernmost limit. In any case, the species has never been common in the Agde and Sète areas, due to the scarcity of hard substrates. In the more southern French Catalonia, eutrophication, particulate organic matter, water turbidity and chemical pollution from run-off from vineyards have been suggested as a cause of the decline of *Cystoseira*^68^. The poor health status of the *Posidonia oceanica* (Linnaeus) Delile seagrass meadows in French Catalonia has also been attributed to this contamination^69^. However, (i) port facilities are uncommon in the area, and no recent man-made structures have artificialized the shoreline^55^; (ii) efficient sewage water treatment plants have been in operation since the 1970–1990s; overall, in the whole area, the water quality of coastal waters and of rivers inputs has greatly improved since the 1990s and is now considered as good to excellent, according to the EU criteria^70^. *Cystoseira mediterranea* is sensitive to heavy N loading^48^, so that pollution could be involved in the decline in the 1960s–1970s, but certainly not that of 2007.

Overgrazing by herbivorous fish and sea urchins is regarded worldwide as a major cause of canopy-forming seaweed regression. Overgrazing is often a cascading consequence of the overfishing of predators of herbivorous organisms. In the western Mediterranean, the teleost *Sarpa salpa* (Linnaeus, 1758) and sea urchins *Paracentrotus lividus* (Lamarck, 1816) and *Arbacia lixula* (Linnaeus, 1758) are the species most involved in overgrazing^14,23,26,27,30,71,72^. *C*. *mediterranea* only thrives in very shallow habitats, less than 0.5–1.0 m depth; as for other shallow seaweed belts, its habitat can be considered as an escape in space, a refuge against grazing^73^. Overgrazing represents therefore an unlikely but possible cause of *C*. *mediterranea* regression. A study on the vicariant species, *C*. *amentacea*, has shown that *Sarpa salpa* was able to graze up to the very shallow infralittoral fringe, and highlighted that fish herbivory pressure could cause the withdrawal of the lower limit of the species^74^. The fluctuations we evidence are probably robust when only the length of the occupied shore is considered, but could be less marked or even smoothed out if the actual surface area is taken into consideration. The recent increase in the abundance of herbivore species^75^ in the area could have resulted in an underestimation of the loss of *C*. *mediterranea*.

In the 1960s–1970s, Gros^68^ described the invasion of the *C*. *mediterranea* habitat by dense stands of the mussel *Mytilus galloprovincialis* (Fig. 4). Mussels could reduce the settlement probability and survival of *C*. *mediterranea* recruits because of the instability caused to the mussels’ support (prone to be pulled out by waves), or to the lack of light for recruits that grow directly on the rocky substrate. Such a negative effect of mussel proliferation on seaweeds has already been reported, e.g. in Sweden^76^. As emphasized by Gros^68^, the proliferation of mussels can be due to organic pollution. However, another hypothesis can be put forward; from the 1950s to the 1970s, mussel farming in France spread considerably, including the development of off-shore farms off Sète. As a result, the number of larvae produced and transported by the Northern Mediterranean Current towards French Catalonia has probably grown. This possibly resulted in an invasion by mussels of the *C*. *mediterranea* habitat. Subsequently, mussel culture has declined, due to massive predation by gilthead seabream (*Sparus aurata* L. 1758) in offshore farms and competition with oyster culture in coastal lagoons, which is more profitable^77^. Consequently, the 1960s–1970s minimum of *C*. *mediterranea* may originate from pollution (see above), but also from this kind of flood of mussel larvae (see^78^, for an invasive mussel).

Finally, the fluctuations of *C*. *mediterranea* populations could also be related to global change, through either the mean Sea Surface Temperature (SST) or exceptional events (heatwaves and storms), that are emerging risks for marine ecosystems^79,80^.

The poleward shift of the range of many species, both seaweeds and metazoans, in parallel with the SST increase, the role of which may be either direct or indirect, is well documented^13,81–83^. Here, the *C*. *mediterranea* withdrawal could seem to occur in the opposite direction to polewards since its northernmost localities became extinct; however, this very local withdrawal is clearly linked to coastal development (see above) rather than to climate warming.

**Figure 5:**
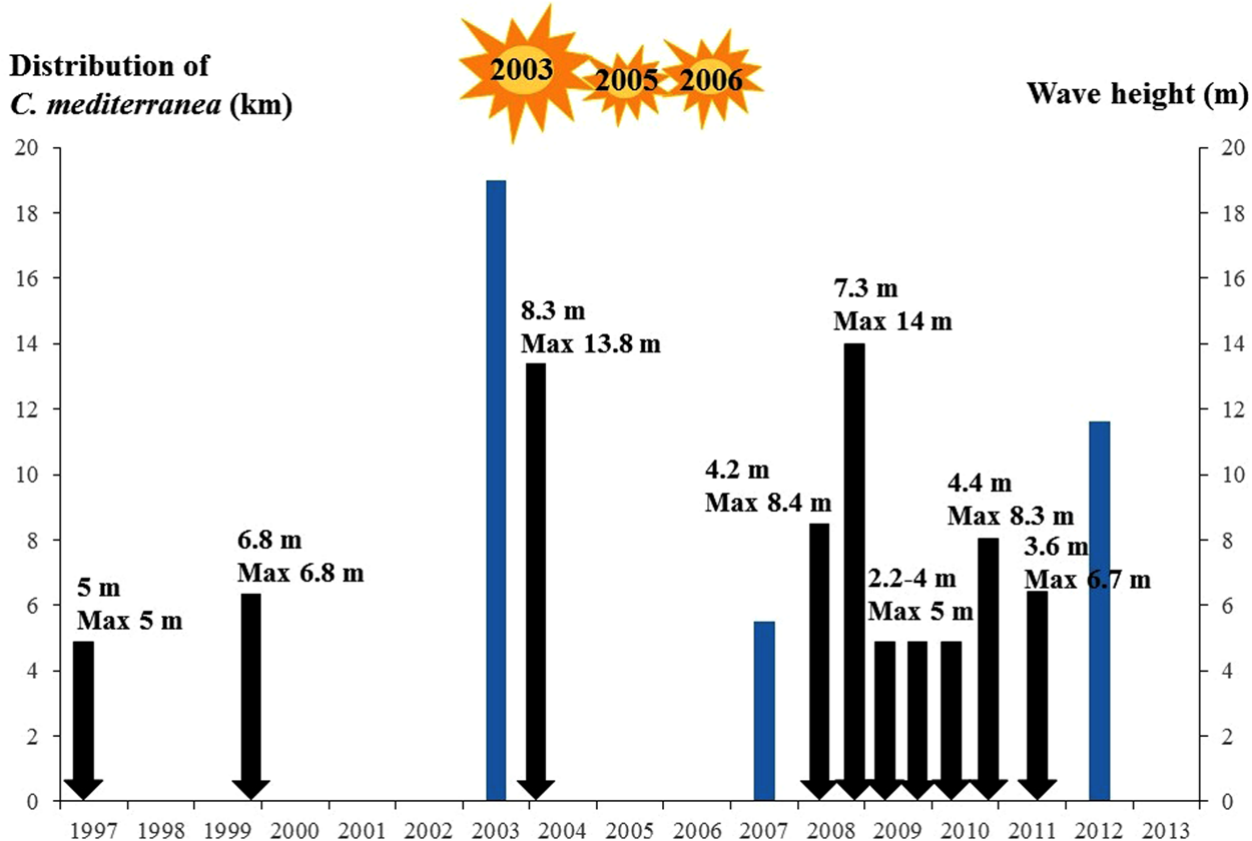
Exceptional storm frequency (black arrows: wave height, mean and maximum) and heatwaves (sun) along the coast of French Catalonia from 1997 to 2012^86,87^. The blue histogram represents the length of rocky coast colonized by *Cystoseira mediterranea* (km). Original drawings.

Two types of exceptional meteorological events have been identified in the study area: exceptional storms and heatwaves (Fig. 5). The first, extreme storms, were recorded in 2003, and since 2008, their frequency has increased substantially. The second type, extreme heatwaves, were recorded in 2003, 2005 and 2006. Rodríguez-Prieto^43^, highlighted the negative effect of a heatwave, coupled with an exceptional low barometric tide (due to the conjunction of two meteorological events: wind and atmospheric pressure), resulting in the long-lasting emersion and mortality of *C*. *mediterranea*. In Spanish Catalonia, the wasting effects of an exceptional extreme storm, down to tens of metres depth, in particular on *Cystoseira* stands, have been described^84,85^. Heatwaves and an exceptional storm are plausible candidates to account for the 2007 decline of *C*. *mediterranea*.

Overall, the following scenario of the fluctuations of *C*. *mediterranea* in French Catalonia can be suggested. A general decline trend has possibly occurred since the turn of the 20^th^ century. It is worth noting that, in the absence of exhaustive surveys similar to those performed since 2003, this decline remains a hypothesis. If it proves true, it could result from the run-off of chemical contaminants from vinyards, the main economic activity of the area. The minimum abundance of the 1960s–1970s, although not accurately measured, is indisputable. It may be related to the general domestic pollution which at that time affected the northwestern Mediterranean basin, and to the flow of mussel larvae originating from mussel culture located upstream. A second minimum, more marked, occurred in 2007. It may be related with three heat waves (inducing mortality during seaweed emersion) and an exceptional storm. The *C*. *mediterranea* individuals, overloaded with mussels, were severely uprooted. This opened up free spaces for recolonization by mussels, which settled directly on the rock, forming patches distinct from those of *C*. *mediterranea* (personal observation of AB and TT). Between 2007 and 2012, further exceptional storms pulled off the mussel beds and opened up space for the recruitment of *C*. *mediterranea* and articulated corallines. *Cystoseira mediterranea* then partially recovered at an unexpected rate.

## Conclusion

The tale of the local extirpation of seaweed populations, of their steady and unidirectional decline, the shift of their range area and the regime shift between canopy-forming species and less structured stands, has been enriched in documentation over the past decades. However, it may be fashionable to describe severe regressions, and less exciting to report moderate regressions, stability or recovery. The present study is a good example of the importance of using long-term data in the study of the variations of Mediterranean marine forests. It highlights the fact that in the Mediterranean Sea, no general conclusions on the decline of marine forests can be drawn. Each species is a particular case that should be studied in a particular area. The present study clearly shows the local extinction of *C*. *mediterranea* in its northern limit and changes over a century of populations located ~70 km westward. In addition, ancient baselines, more than a century old, are poorly or not accurately known. Cases of moderate decline, or decline followed by natural re-colonization, are known, although often overlooked by authors when they write the introduction of an article on marine seaweed forests. The reports of natural or human-driven fluctuations, decline and recovery usually deal with relatively short-lived species.

Because of the rare opportunity of an unusually long observation period, ~120 years, in a well-explored coastline area, we were able to show that sharp fluctuations in density and occupied shore length, and natural recovery episodes, occurred over time, reflecting a higher than expected resilience and a health status that is better than that reported for many canopy-forming seaweeds of the world ocean. In addition, we provided baselines for future surveys with a very high standard of accuracy.

## Supplementary information

Table S1
