# Supplementary material for: The ups and downs of a canopy-forming seaweed over a span of more than one century
Source: Sci Rep. 2019 Mar 27;9:5250. doi: 10.1038/s41598-019-41676-2 (PMC6437179; doi:10.1038/s41598-019-41676-2)
Supplement: Supplementary file 1 — Table S1 [file 41598_2019_41676_MOESM1_ESM.pdf]

Title: The ups and downs of a canopy-forming seaweed over a span of more than one century

Aurélie Blanfuné, Charles François Boudouresque, Marc Verlaque, Thierry Thibaut

Table S1. Records of *C. mediterranea* along *Occitanie* Region and French Catalonia (Gulf of Lions, South in France). 'Town' means '*commune*', the smallest administrative district in France, either a town or a village. 'Location' means the name of the place, as reported by the author(s); it can be a large area (hundreds of kilometers), or a tiny hamlet, locatable or not on a current map; the authors' spelling of the locality has been respected, even if it has changed today; the authors' name of the locality is respected, even if it is obviously misapplied (e.g. 'Montpellier', which is not a coastal city). The names of the herbaria, the texts handwritten by the collector on the voucher and the texts written in the publications were not translated into English, where English was not the original language (columns 'Location' and 'Reference').

French Catalonia

**Year**	**Town**	**Location**	**References**
19th	Banyuls	Banyuls	Sauvageau *in* Hamel, 1931-1939
19th	Banyuls	Banyuls	Sauvageau *in* Hamel, 1931-1939, as *Cystoseira mediterranea* var. *valiantei*
19th	Collioure	Collioure	Herbier Lamouroux, Fucacées, MS63p018, PC011473, C1f65, Muséum national d'histoire naturelle de Paris (PC)
19th	Port-Vendres	Port-Vendres	Sauvageau *in* Hamel, 1931-1939, as *Cystoseira mediterranea* var. *valiantei*
19th	Port-Vendres	Port-Vendres	Sauvageau *in* Hamel, 1931-1939
1818	Port-Vendres	Port-Vendres	Blateau *in* Herbier Général, Classeur 42, Herbier Montpellier Université (MPU)
1883	Collioure	Saint Llatzaire, un peu en dessous du niveau moyen dans les zones battues	Herbier Flahault, Classeur 31, C1-31, Herbier Montpellier Université (MPU)
1883	Collioure	Rochers Saint llatzer, Collioure	Herbier Flahault, Classeur 32, Herbier Montpellier Université (MPU)
1883	Collioure	Collioure	Herbier Flahault, Classeur 32, Herbier Montpellier Université (MPU)
1883	Collioure	Collioure	Sauvageau *in* Herbier Flahault, Classeur 33, Herbier Montpellier Université (MPU)
1883	Collioure	Sous la flarelle, Collioure	Herbier Flahault, Classeur 33, Herbier Montpellier Université (MPU)
1905	Banyuls	Banyuls	Herbier Sauvageau SA4511, Muséum national d'histoire naturelle de Paris (PC), as *Cystoseira mediterranea* Sauv.
1906	Banyuls	Banyuls	Herbier Sauvageau SA4433, Muséum national d'histoire naturelle de Paris (PC), as *Cystoseira mediterranea* Sauv.

1906	Banyuls	Banyuls	Herbier Sauvageau SA4435, Muséum national d'histoire naturelle de Paris (PC), as *Cystoseira mediterranea* Sauv.
1906	Banyuls	Banyuls	Herbier Sauvageau SA4436, Muséum national d'histoire naturelle de Paris (PC), as *Cystoseira mediterranea* Sauv.
1906	Banyuls	Banyuls	Herbier Sauvageau SA4438, Muséum national d'histoire naturelle de Paris (PC), as *Cystoseira mediterranea* Sauv.
1906	Banyuls	Banyuls	Herbier Sauvageau SA4466, Muséum national d'histoire naturelle de Paris (PC), as *Cystoseira mediterranea* Sauv.
1906	Banyuls	Banyuls	Herbier Sauvageau SA4471, Muséum national d'histoire naturelle de Paris (PC), as *Cystoseira mediterranea* Sauv.
1906	Banyuls	Banyuls	Herbier Sauvageau SA4480, Muséum national d'histoire naturelle de Paris (PC), as *Cystoseira mediterranea* Sauv. LECTOTYPE
1906	Banyuls	Banyuls	Sauvageau *in* Herbier Flahault, Classeur 33, Herbier Montpellier Université (MPU)
1906	Banyuls	Banyuls	Savageau *in* Herbier Flahault, Classeur 33, Herbier Montpellier Université (MPU)
1906	Banyuls	Banyuls	Sauvageau *in* Herbier Flahault, Classeur 33, Herbier Montpellier Université (MPU)
1906	Banyuls	Banyuls	Herbier Sauvageau, Algues de la Méditerranée, PC0519085, SA4299, Muséum national d'histoire naturelle de Paris (PC), as *Cystoseira ericoides*
1906	Port-Vendres	Port-Vendres	Sauvageau *in* Herbier Flahault, Classeur 33, Herbier Montpellier Université (MPU)
1907	Banyuls	Banyuls	Sauvageau *in* Herbier Mouret, Algue de Provence II, part n°71, Muséum d'histoire naturelle de Toulon (TLON)
1907	Banyuls	A droite de la jetée, Banyuls	Sauvageau *in* Herbier Flahault, Classeur 33, Herbier Montpellier Université (MPU)
1907	Banyuls	Banyuls, Cap Doune	Sauvageau *in* Herbier Villa Thuret, Liasse 282 - *Cystoseira*, planche 283, Jardin Botanique de la Villa Thuret (VTA)
1907	Banyuls	Cap Doune	Sauvageau *in* Herbier Flahault, Classeur 33, Herbier Montpellier Université (MPU)
1907	Banyuls	Cap Doune	Sauvageau *in* Herbier Flahault, Classeur 33, Herbier Montpellier Université (MPU)
1907	Banyuls	Baie du Troc, Banyuls	Sauvageau *in* Herbier Flahault, Classeur 33, Herbier Montpellier Université (MPU)
1907	Banyuls	Banyuls	Sauvageau *in* Herbier Flahault, Classeur 33, Herbier Montpellier Université (MPU)
1907	Banyuls	Banyuls	Sauvageau *in* Herbier Flahault, Classeur 33, Herbier Montpellier Université (MPU)
1907	Banyuls	Banyuls	Sauvageau *in* Herbier Flahault, Classeur 33, Herbier Montpellier Université (MPU)

1907	Banyuls	Banyuls	Sauvageau *in* Herbier Villa Thuret, Liasse 282(2) - *Cystoseira*, planche 283, Jardin Botanique de la Villa Thuret (VTA)
1907	Banyuls	Banyuls	Sauvageau *in* Herbier Villa Thuret, Liasse 282 - *Cystoseira*, planche 283, Jardin Botanique de la Villa Thuret (VTA)
1907	Banyuls	Baie du Troc, Banyuls	Sauvageau *in* Herbier Flahault, Classeur 33, Herbier Montpellier Université (MPU)
1907	Banyuls	A gauche de la jetée, Banyuls	Sauvageau *in* Herbier Flahault, Classeur 33, Herbier Montpellier Université (MPU)
1907	Banyuls	Cap Dosne	Sauvageau *in* Herbier Flahault, Classeur 33, Herbier Montpellier Université (MPU)
1907	Banyuls	Cap Dosne	Sauvageau *in* Herbier Flahault, Classeur 33, Herbier Montpellier Université (MPU)
1907	Banyuls	Banyuls	Herbier Sauvageau SA4486, Muséum national d'histoire naturelle de Paris (PC), as *Cystoseira mediterranea* Sauv.
1907	Banyuls	Banyuls	Herbier Sauvageau SA4490, Muséum national d'histoire naturelle de Paris (PC), as *Cystoseira mediterranea* Sauv.
1907	Banyuls	Banyuls	Herbier Sauvageau SA4481-SA4485, Muséum national d'histoire naturelle de Paris (PC), as *Cystoseira mediterranea* Sauv.
1907	Banyuls	Banyuls	Herbier Sauvageau SA4487-SA4489, Muséum national d'histoire naturelle de Paris (PC), as *Cystoseira mediterranea* Sauv.
1907	Banyuls	Banyuls	Herbier Sauvageau SA4512-SA4422, Muséum national d'histoire naturelle de Paris (PC), as *Cystoseira mediterranea* Sauv.
1909	Banyuls	Banyuls	Sauvageau *in* Herbier Flahault, Classeur 33, Herbier Montpellier Université (MPU)
1909	Collioure	De Collioure à Port-Vendres	Herbier E.J. Neyraut *in* Herbier Général, Classeur 44, Pochette *C. mediterranea*, Herbier Montpellier Université (MPU)
1909	Port-Vendres	Port-Vendres	E.J. Neyraut *in* Herbier Général, Classeur 42, Herbier Montpellier Université (MPU)
1910	Banyuls	Banyuls	Sauvageau *in* Herbier Flahault, Classeur 33, Herbier Montpellier Université (MPU)
1910	Banyuls	Banyuls	Sauvageau *in* Herbier Flahault, Classeur 33, Herbier Montpellier Université (MPU)
1910	Banyuls	Banyuls	Sauvageau *in* Herbier Flahault, Classeur 33, Herbier Montpellier Université (MPU)
1910	Banyuls	Banyuls	Sauvageau *in* Herbier Flahault, Classeur 33, Herbier Montpellier Université (MPU)
1910	Banyuls	Musoir	Herbier Sauvageau, Algues de la Méditerranée, PC0519079, SA4301, Muséum national d'histoire naturelle de Paris (PC), as *Cystoseira ericoides*
1911	Banyuls	Banyuls	Sauvageau *in* Herbier Flahault, Classeur 33, Herbier Montpellier Université (MPU)

1913	Banyuls	Rochers de l'Ile Grosse, Banyuls, Pyrénées orientales	Herbier de Raymond Gousme *in* Herbarium Universitatis Conimbrigensis Leg, Det. P. Hariot, COI 3866, planche venant de l'Herbarium cryptogamique du Muséum national d'histoire naturelle de Paris, Herbarium of University of Coimbra (COI)
1926	Banyuls	Banyuls	Raphèlis *in* Herbier Général, Classeur 42, Herbier Montpellier Université (MPU)
1926	Banyuls	Banyuls	Herbier Raphèlis *in* Herbier Général, Classeur 44, Pochette *C. mediterranea*, Herbier Montpellier Université (MPU)
1932	Banyuls	Banyuls	Leg Hamel-Joukov, det Feldmann *in* Herbier Algues de France non trié, Fascicule IV, 15 planches, n°181, Muséum national d'histoire naturelle de Paris (PC)
1932	Banyuls	Banyuls	Exsiccata Hamel, Algues de France, n°181, Leg Hamel - Joukov, det. Feldmann, Herbier Montpellier Université (MPU)
1934	Banyuls	Banyuls	Herbier Feldmann *in* Herbier Roger Melin, liasse Algues marines, Muséum national d'histoire naturelle de Paris (PC)
1937	Banyuls	Cap l'Abeille	Feldman, 1937
1937	Banyuls	Cap Béar	Feldman, 1937
1937	Banyuls	Cap du Troc	Feldman, 1937
1937	Banyuls	Cap d'Osne	Feldman, 1937
1937	Banyuls	île Grosse	Feldman, 1937
1937	Banyuls	Cap l'Abeille	Feldmann, 1937, as *Cystoserira mediterranea* var. *valiantei*
1937	Cerbère	Cap Cerbère	Feldman, 1937
1937	Collioure	Collioure	Feldman, 1937
1937	Port-Vendres	Port-Vendres	Feldman, 1937
1950	Banyuls	Anse du Troc, Banyuls	Herbier Roger Melin, liasse Algues marines, Muséum national d'histoire naturelle de Paris (PC)
1953	Banyuls	Banyuls	Herbier Magne (Planches + Carnet de Récolte), n°262, Muséum national d'histoire naturelle de Paris (PC)
1955	Banyuls	ile grosse	Blackler, 1955
1962	Banyuls	Banyuls	D. Gallant *in* Herbier N et F. Hallé, Herbier Montpellier Université (MPU)
1967	Banyuls	Face au laboratoire de Banyuls	Herbier Meinesz, Classeur Fucales *Cystoseira* Med, part n°MPCYS0980, E.A. 4228 ECOMERS Université de Nice Sophia Antipolis
1967	Banyuls	Face au laboratoire Arago, au niveau	Herbier Meinesz, MPCY0980, E.A. 4228 ECOMERS Université de Nice Sophia Antipolis
1968	Banyuls	Banyuls	Pellegrini and Pellegrini, 1970
1969	Banyuls	Cap du Troc	Boudouresque, 1969
1969	Banyuls	Cap du Troc	Boudouresque, 1971

1970	Banyuls	Cap l'Abeille	Boudouresque, 1970
1970	Banyuls	Cap l'Abeille	Boudouresque, 1970
1972	Côtes des Albères	Côtes des Albères	Boudouresque, 1972
1996	Banyuls	Banyuls	Charles *et al.*, 1996

Languedoc

Year	Town	Location	References
19th	Sète	Cette	Sauvageau *in* Hamel 1931-1939, as *Cystoseira mediterranea* var. *valiantei*
19th	Sète	Cette	Herbier Général, AB6345, PC0527470, Muséum national d'histoire naturelle de Paris (PC), as *Cystoseira ericoides*
19th	Sète	Cette	Herbier Montagne, liasse 2 - *Cystoseira* 2, MA7728, PC043582, Muséum national d'histoire naturelle de Paris (PC), as *Cystoseira ericoides*
19th	Sète	Cette	Herbier Général, AB6345, PC0527470, Muséum national d'histoire naturelle de Paris (PC), as *Cystoseira ericoides*
19th	Sète	Cette	Don de Laveaux *in* Herbier Général, AB6325, PC0527484, Muséum national d'histoire naturelle de Paris (PC), as *Cystoseira ericoides*
19th	Sète	Cette	Roubieux *in* Herbier J.V.F. Lamouroux, PC0111494, Muséum national d'histoire naturelle de Paris (PC), as *Cystoseira ericoides*
19th	Sète	Sète	Sauvageau *in* Hamel, 1931-1939
1817	Agde	Agde, vis-à-vis le fort de Brescoui	Herbier J.V.F. Lamouroux, Fucacées, PC0111478, C1f69, n°166, Muséum national d'histoire naturelle de Paris (PC)
1817	Villeneuve-lès-Maguelone	Plage de Maguelonne, Montpellier	Herbier J.V.F. Lamouroux, Fucacées, PC0111479, C1f569, n°167, Muséum national d'histoire naturelle de Paris (PC), as *Fucus selaginoides*
1818	Maguelonne	Maguelonne	Roubieux *in* Herbier J.V.F. Lamouroux, PC0111600, Muséum national d'histoire naturelle de Paris (PC), as *Fucus selaginoides*
1824	Languedoc	Côtes méditerranéennes du Languedoc	J.V.F. Lamouroux *in* Herbier Bory de Saint-Vincent, TA7943, PC0594772, Muséum national d'histoire naturelle de Paris (PC), as *Fucus selaginoides*
1890	Sète	Cette, rejetée par la tempête sur la plage en face des salins de Villeroy	Herbier E.J. Neyraut *in* Herbier Général, det. C. Sauvageau, Classeur 44, Pochette *C. mediterranea*, Herbier Montpellier Université (MPU)
1890	Sète	Cette, rejetée par la tempête sur la plage en face des salins de Villeroy	Herbier E.J. Neyraut *in* Herbier Général, det. C. Sauvageau, Classeur 44, Pochette *C. mediterranea*, Herbier Montpellier Université (MPU)

1910	Sète	Brise Lame de Cette	Sauvageau *in* Herbier Flahault, Classeur 33, Herbier Montpellier Université (MPU)
1910	Sète	Brise Lame de Cette	Savageau *in* Herbier Flahault, Classeur 33, Herbier Montpellier Université (MPU)
1920	Sète	Cette, rejeté sur la plage par la tempête	Herbier E.J. Neyraut *in* Herbier Général, Classeur 44, Pochette *C. mediterranea*, Herbier Montpellier Université (MPU)
1982	Agde	La Grande Conque, peu fréquente	Lauret, 1982
1982	Agde	Cap d'Agde	Satra, 1998
1982	Agde	Cap d'Agde	Richard, 1999

References

Blackler, H. Pre – Congress phycological excursion to the Côte des Alberes Banyuls-sur-mer, June 22 nd – 29th 1954. *Brit. Phycol. J.* **1 (3)**, 15-18 (1955). 10.1080/00071615500650041 (1955).

Boudouresque, C.F. Etude qualitative et quantitative d'un peuplement algal à *Cystoseira mediterranea* dans la région de Banyuls-sur-Mer. *Vie Milieu.* **20 (2B)**, 437-452 (1969).

Boudouresque, C.F. Recherches de bionomie analytique, structurale et expérimentale sur les peuplements benthiques sciaphiles de Méditerranée occidentale (fraction algale). *Thèse de Doctorat. Université de Marseille.* 1-624 (1970).

Boudouresque, C.F. Recherches de bionomie analytique, structurale et expérimentale sur les peuplements benthiques sciaphiles de Méditerranée occidentale (fraction algale). La sous-strate sciaphile des peuplements de grandes *Cystoseira* de mode battu. *Bull. Mus. Hist. Nat. Marseille*. **31**, 141-151 + 1 Tabl. (1971).

Boudouresque, C.F. Recherches de bionomie analytique structurale et expérimentale sur les peuplements benthiques sciaphiles de Méditerranée occidentale (fraction algale) : biotopes sciaphiles et stations étudiées. *Bull. Mus. Hist. Nat. Marseille*. **32**, 177-188 (1972).

Charles, F., Grémare, A. & Amouroux, J.M. Ingestion rates and absorption efficiencies of *Abra ovata* (Mollusca: Bivalvia) fed on macrophytobenthic detritus. *Estuar. Coast. Shelf S.* **42**, 83-102 (1996).

Feldmann, J. Recherches sur la végétation marine de la Méditerranée. La Côte des Albères. *Rev. Algol.* **10**, 1-339 (1937).

Hamel, G. Phéophycées de France, Imprimerie Wolf, Rouen, XLVI, 1- 431 (1931-1939).

Lauret, M. Les Macroalgues du Cap d'Agde. *113e Session extraordinaire de la société Botanique de France.* 59-75 (1982).

Pellegrini, L. & Pellegrini, M. Sur d'éventuelles possibilités d'exploitation de quelques Cystoseires méditerranéennes. *Ann. Soc. Sci. Nat. Arch. Toulon Var*. **22**, 133-138 (1970).

Richard, E. Etude de la Biodiversité des macroalgues du Cap d'Agde. *Mémoire de Maîtrise de Biologie des populations et des écosystèmes. Université Montpellier II Sciences et Techniques du Languedoc, France.* 1- 15 + annexes 1-6 (1999).

Satra, F. Etude de la biodiversité des algues du Cap d'Agde- Février-Avril 1998. *Mémoire de Maîtrise, Université de Montpellier II Sciences et Techniques du Languedoc, France*. 1-11 + annexes 1-6 (1998).
